# Targeting Metabolic Syndrome with a Pre-Conception True-Couples-Based Lifestyle Intervention: A Pre-Post Mixed-Methods Evaluation

**DOI:** 10.3390/nu17122037

**Published:** 2025-06-18

**Authors:** Sundus Nizamani, Catherine R. Knight-Agarwal, Li Li, Alexandria N. Mekanna, Rosemary Anne McFarlane

**Affiliations:** 1 Discipline of Public Health, Health Research Institute, Faculty of Health, University of Canberra, Canberra, ACT 2617, Australia; ro.mcfarlane@canberra.edu.au; 2 Nutrition & Dietetics, Faculty of Health, University of Canberra, Canberra, ACT 2617, Australia; cathy.knight-agarwal@canberra.edu.au; 3 School of Science, Western Sydney University, North Parramatta, NSW 2151, Australia; l.li7@westernsydney.edu.au (L.L.); 20641926@student.westernsydney.edu.au (A.N.M.)

**Keywords:** couples, metabolic syndrome, preconception, diet, physical activity

## Abstract

Background/Objectives: Metabolic syndrome (Mets) risk is influenced by both parents’ preconception lifestyle, yet most interventions target individuals rather than couples. True couples-based interventions that engage both partners equally remain rare. This study aimed to assess the feasibility and adherence of a 10-week lifestyle intervention delivered to heterosexual couples in the preconception period. Methods: This was a pre-post mixed-methods study involving eight nulliparous, cohabiting couples (N = 16 participants) planning a pregnancy within three years. Couples received tailored dietary and physical activity advice via remote sessions. Qualitative data were collected through post-intervention dyadic interviews and thematically analysed to explore participants’ experiences and perspectives on feasibility and adherence. Quantitative data on anthropometry, dietary intake (serves from five food groups), and sedentary behaviour were descriptively analysed. Wilcoxon signed-rank tests were used to assess changes in paired outcomes. Results: qualitative findings highlighted shared motivation, mutual accountability, cultural barriers, and the practicality of the intervention structure. All couples completed the intervention (100% retention). Among participants who required change, improvements were observed in all eight individuals for body mass index and in five out of seven individuals for waist-to-hip ratio. Statistically significant improvements were found in BMI (*p* = 0.027) and grain intake (*p* = 0.002), while other dietary and anthropometric changes were not significant. Dietary improvements were noted in 43 out of 80 observations across vegetables, fruits, grains, protein, and dairy intake. Sedentary hours were reduced in 12 of 16 participants, though increases in physical activity intensity were limited. Conclusions: A true-couples-based lifestyle intervention is feasible and acceptable in the preconception period. The approach shows potential for improving diet and reducing sedentary behaviour. Future research with a larger sample and longer duration is recommended to assess long-term effectiveness and broader applicability.

## 1. Introduction

Worldwide, metabolic syndrome (Mets) is a significant public health issue [1]. It is a major risk factor for non-communicable diseases (NCDs) such as type 2 diabetes, cardiovascular disease, and certain cancers [2,3]. Defined by a cluster of conditions—abdominal adiposity, hypertension, insulin resistance, and dyslipidaemia—it not only affects individual health but also places substantial demands on healthcare systems. These burdens lead to substantial financial costs [4]. For example, in 2024, the three countries with the highest obesity-related economic burdens were estimated to have lost billions of US dollars due to obesity alone [4]. Mets can be transmitted via lifestyle as well as epigenetic mechanisms from both the mother and the father [5,6], making it essential to address the health and lifestyle choices of both parents prior to conception. The preconception period offers a valuable opportunity for lifestyle changes, with evidence suggesting potential long-term benefits in improving dietary habits and reducing metabolic risk [7].

Managing weight and increasing physical activity are established strategies for reducing Mets risk [8,9,10]. However, adherence to these behaviours is challenging due to socio-ecological factors, psychological barriers, and a lack of social support, which often impact adherence [11,12,13]. Nevertheless, a systematic review found that couples participating together in health interventions tend to have higher adherence rates and are more likely to sustain lifestyle changes over time, as the presence of a supportive partner reinforces motivation, mutual accountability, and shared commitment to health goals [14]. Moreover, spousal support in health programs, such as weight loss interventions, enhances commitment and outcomes, often surpassing the effects of partner-supported models where only one partner is the primary target [15,16,17]. Dietary modifications based on national guidelines, such as the Australian Guide to Healthy Eating (AGHE), play a crucial role in improving metabolic health and reducing Mets risk [18]. The AGHE promotes a balanced intake of nutrient-dense foods, including high-fibre whole grains, lean proteins, and healthy fats, which have been shown to improve insulin sensitivity, lower blood pressure, and reduce central adiposity—key factors in Mets [19]. Furthermore, adherence to such dietary patterns is higher when individuals engage in lifestyle changes alongside their partners, reinforcing dietary accountability and shared meal planning strategies that support long-term adherence [16].

Despite this evidence, most existing studies focus on partner-supported models, where one member of the couple is the primary participant while the other plays a secondary, supportive role [15,16,17]. True couples-based interventions (TCBI) are rare and, in contrast, are designed to engage both partners equally as active participants rather than assigning one a supportive role [14]. Such approaches hold the potential to enhance mutual accountability, improve adherence, and provide greater health benefits compared to one-sided approaches [14,17]. By involving both partners equally, these interventions could also mitigate the risk of passing Mets-related vulnerabilities to future generations by targeting both members of a couple in interventions to improve preconception health and monitoring the progress of both, rather than just one.

In response to this gap in the literature, the present study explores the feasibility of a true couples-based lifestyle intervention targeting diet and physical activity to reduce Mets risk. By involving both members of a couple, it examines the impact of joint participation on adherence, motivation, and outcomes. Additionally, it addresses practical considerations such as recruitment, retention, and engagement, laying the groundwork for more comprehensive future interventions.

## 2. Methods

### 2.1. Design of the Study

A non-randomised, single-arm mixed-methods study was designed using an exploratory sequential mixed-methods approach [20]. The initial qualitative phase [21] provided rich, in-depth insights that enabled the co-design of the intervention, tailoring it to participant experiences and needs. This phase identified key areas related to mutual support and shared engagement in health goals. For example, participants stressed the value of joint activities, such as shared meal planning and physical activity sessions, which were incorporated into the intervention’s structure to support couples in working collaboratively. These adjustments were designed to reinforce a supportive environment for both partners, encouraging sustained adherence to dietary and physical activity changes throughout the program. Following this, the current study used these insights to evaluate the feasibility and effectiveness of the intervention. The comprehensive evaluation focused on both individual and couple-level dynamics, helping to identify logistical and methodological considerations. Ultimately, the study aimed to explore the potential of a preconception TCBI to promote healthy lifestyle changes.

The study was approved by the Human Research Ethics Committee (No. 10424). Informed written consent was obtained from all participants. Before beginning the intervention, each couple attended an information session where they were briefed on the study’s objectives, procedures, and their roles as participants.

### 2.2. Sampling and Inclusion/Exclusion Criteria

Couples were recruited for the study through multiple channels, including university announcements, community outreach, and online platforms in Australia. Couples were required to be nulliparous, cohabiting, aged between 21 and 45 years, planning a pregnancy within the next three years, heterosexual, with both partners providing informed consent. Exclusion criteria included being currently pregnant or having children, not living together, a history of bariatric surgery, ongoing use of assisted reproductive technologies, an obesity index above 38 kg/m^2^, or any co-morbid condition such as hypertension or cancer that would require clinical intervention outside the scope of this lifestyle-focused study. Recruitment was limited to heterosexual couples to allow exploration of epigenetic transmission from both biological parents.

### 2.3. The Intervention

**Figure 1:**
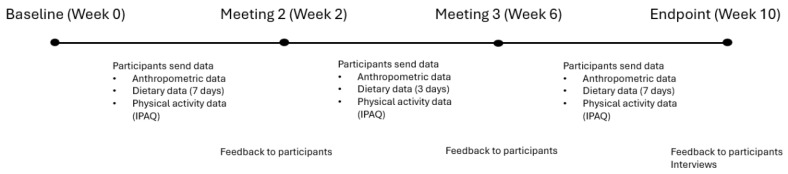
Timeline and structure of the 10-week couples-based lifestyle intervention, including data collection points, feedback sessions, and behaviour change techniques.

The intervention was structured as a 10-week program conducted between September 2022 to December 2023, designed to encourage healthier lifestyle behaviours in couples. The length of the intervention was supported by literature where epigenetic changes were observed in 8 to 10 weeks [22,23,24,25]. The couples met with SN three times over Zoom meetings, in addition to an introductory meeting where they learnt the components of the program. The program components focused on self-monitoring, structured reporting, and support activities that engaged both members of each couple. Quantitative data collection occurred at two designated times: baseline and endpoint. An additional mid-point data collection occurred for diet diaries and physical activity (IPAQ) [26]. Qualitative interviews were conducted upon completion of the intervention. A 12-month rolling recruitment approach was used with staggered start dates which allowed each couple to proceed through the intervention based on individual availability. Figure 1 provides a visual overview of the intervention structure, including the timing of data collection, feedback sessions, and embedded behaviour change techniques such as self-monitoring and personalised feedback.

## 3. Data Collection and Analysis

### 3.1. Outcome Measures

The study examined variables hypothesised to influence the intervention’s effectiveness and its impact on Mets risk. Details of feasibility and adherence measures and anthropometric, dietary, and physical activity measures and methods of measurements are provided in Table 1.

### 3.2. Qualitative Data

Participants were asked about their reasons for participation in the study on the sign-up form prior to commencement. Upon completion of the intervention semi-structured dyadic interviews [27] were conducted via Zoom to explore their experiences with the intervention and how they engaged with the health behaviour changes. Both members of a couple were interviewed at the same time, and the length of the interview was approximately one hour per couple. The interview guide was developed based on the study objectives and informed by themes identified during the design phase of the intervention. Mock interviews were conducted to test and refine the questions for clarity and relevance. The guide was further shaped by insights from the existing literature [27,28] and informed by approaches to dyadic interview design in couples-based research [29], as well as examples drawn from the previous qualitative study [21], which provided a foundation for exploring participants’ experiences. The interviews covered multiple topics, including the experiences of implementing dietary and physical activity changes and the dynamics of working together as a couple to meet shared health goals. These interviews provided insight into how couples approached and maintained health behaviours together, as well as any challenges or supports that influenced their engagement with the program. During the interview process, SN was mindful to remain neutral so as not to influence participant responses.

A thematic analysis [30,31] was then undertaken with NVivo software (version 12) [32] and Excel. SN developed initial codes through an iterative process, with these codes then clustered into themes that reflected the study’s objectives. Assistance with this process was provided by CKA and RM, serving as an important form of triangulation, with regular discussions facilitating agreement of the final set of themes. Quotes embedded in the results were given a unique identifier to connect participants.

Integration also occurred during data analysis, where health-related outcomes from the quantitative data were contextualised with insights gained from participant interviews. This integrated approach aimed to capture a holistic understanding of the intervention’s feasibility, linking quantitative data with participants’ lived experiences. Adherence was tracked quantitatively through completion rates at each data collection point and qualitatively through dyadic interviews exploring engagement and mutual support. By combining quantitative and qualitative data, the study sought to provide a comprehensive view of the couples-based intervention, focusing on both practical feasibility and participant engagement.

### 3.3. Quantitative Data

Quantitative data analysis focused on comparing pre- and post-intervention results to assess changes in body mass index (BMI), waist-to-hip ratio, physical activity levels, and dietary intake. This analysis also contributed to assessing the feasibility of the intervention by determining the practicality of participants completing measurements and diaries, as well as the potential impact of the intervention on targeted health behaviours. Descriptive statistics were used to present the raw differences between baseline and endpoint measures, avoiding the use of medians or interquartile ranges to align with study objectives. Additionally, the Wilcoxon Signed-Rank Test, a non-parametric statistical method [33], was employed to evaluate paired differences in pre- and post-intervention data. This test was selected for its suitability with small sample sizes and non-normally distributed data, allowing for robust comparison of changes in anthropometric and dietary intake variables.

Software tools such as FoodWorks 10 facilitated dietary intake analysis commonly used by local nutrition professionals [34]. The food composition databases used were compiled based on AUSNUT 2011–12 [35], a food composition database used in the dietary data collection application Easy Diet Diary (version 6.0.28) [36], IPAQ responses were processed using Dr Cuisle Forde’s guide to scoring the IPAQ [37] to gauge physical activity levels, RStudio (version 2024.04.1) and R software (version 4.4.1) supported the overall statistical analysis of anthropometric data [38,39]. Adherence was also monitored quantitatively through session attendance and completion rates at each time point (Baseline, midpoint and endpoint).

Anthropometric measurements included weight, BMI, and waist-to-hip ratio. Each couple was provided with a Lufkin W606PM tape (Crescent Lufkin, Saginaw, MI, USA) to measure waist and hip circumferences. Waist-to-hip ratio is a strong indicator for chronic illnesses [40,41,42,43,44]. Height and weight were self-reported, and BMI was calculated as body weight (kg) divided by the square of the height (m). At the initial meeting, participants were given both a verbal explanation and a copy of the International Standards for Anthropometric Assessment [45] to guide accurate anthropometric self-measurement. Participants used their own weighing scales to measure weight. Recognising the potential variability between different weighing scales, the progress was not compared between couples.

Dietary intake was collected via the Easy Diet Diary app (version 6.0.28) [36] and assessed using FoodWorks 10 [34]. Self-reported estimated food diaries were collected for seven consecutive days both at baseline and at the end point, in addition to a three-day food diary (two working days and one weekend day) at the mid-point. At the initial screening visit, couples were provided with a demonstration of how to use the Easy Diet Diary app (version 6.0.28) plus instructions on how to measure food and fluid intake according to standard metric measurements. Once completed, the participants sent their food diaries to SN, who first checked for accuracy and completeness. Data were entered into FoodWorks 10 [34] for dietary analysis before sending through to the dietitian (CKA, ANM) for interpretation. Personalised advice for each member of a couple, was then curated based on the data provided. This advice was relayed verbally by SN in a meeting, and the report was sent via email (Supplementary File S1).

The primary focus of dietary analysis was on the number of serves consumed from various food groups, including fruits, vegetables, grains, proteins, and dairy, in accordance with the Australian Guide to Healthy Eating [46]. Emphasis was placed on assessing whether participants improved their intake of these core food groups, aiming to observe progress toward meeting dietary guidelines [46] over the course of the intervention.

Physical activity levels were monitored using the International Physical Activity Questionnaire (IPAQ) [26] with training provided on the Borg’s scale of Rate of Perceived Exertion [47], which were self-administered at baseline, mid-point, and endpoint. The IPAQ has been validated in adults across multiple countries, including Australia, demonstrating acceptable reliability and validity for assessing physical activity levels [26]. During the baseline meeting, the IPAQ was explained to participants to ensure accurate completion. The IPAQ was used to obtain a weekly summary of physical activity across multiple domains, such as work, transportation, and leisure. Given the link between sedentary behaviour and Mets [48], the intervention placed particular emphasis on tracking and documenting changes in time spent in sedentary activities. These tools allowed for a detailed view of participants’ physical activity patterns and potential shifts in sedentary behaviour throughout the intervention.

## 4. Results

Sixteen participants (eight couples) were recruited and completed the intervention. Participant details are presented in Table 2. Participants ranged in age from 23 to 42 years and were either married, engaged, or in long-term committed relationships. Relationship lengths varied, with some couples together for 2–4 years and others for 5 years or more.

Participants were from diverse cultural and demographic backgrounds. Languages spoken at home included Bangla, Bahasa, English, Cantonese, Telugu, and Urdu.

### 4.1. Participant Motivations

Participants had varied motivations for joining the study, as documented in the sign-up form (see Table 2). Some aimed to improve their general health and fitness or lose weight (e.g., F1, M4). Others expressed a desire to prepare for a healthy pregnancy (e.g., F1, M1). Additional motivations became more evident through the interviews, including the value of receiving personalised advice. For example, M2 said, “Getting feedback helps us see what we are missing in our diets.” Similarly, M3 reported, “The interim review was helpful in reminding me of things I knew, like not eating enough vegetables.” Most couples also spoke about shared health goals related to improving diet and increasing physical activity. According to F2, “Doing it with someone else definitely encourages you.” F7 echoed this by saying, “If it was just me, I wouldn’t have tried very hard. Having accountability from another person really helps with things like this.” Similar sentiments were expressed by F1, who shared, “When I was not feeling to follow it properly, he was encouraging me: ‘Okay, let’s do it.’”

### 4.2. Feasibility of the Intervention

Feasibility was evaluated based on recruitment, retention, and the practicality of delivering the intervention to both members of a couple (Table 1). All couples successfully enrolled and remained in the study, completing each stage of the intervention. The structure requiring self-measurement of anthropometric variables, food intake, and physical activity was manageable for participants, suggesting that delivery to couples in this format is practical and acceptable. Monitoring tools were perceived as easy to use and helpful, with M1 explaining, “I think the app made everything easier for us. Otherwise, we couldn’t count the calories. The app showed when we were crossing the limit, helping us keep it under control.” Dietary tracking was also described as straightforward, with F2 noting, “The app was pretty much the easiest part of the program,” and F8 adding, “Honestly, I was just logging things. The diet plan you provided wasn’t hard to follow.” Physical activity tracking was considered manageable as well; for example, F4 shared, “Filling in the form reminded me that I need to do more,” and M5 stated, “It wasn’t difficult… We knew what we were doing, and it was common for us.” These reflections support the feasibility of self-monitoring as part of the intervention. Additionally, the staggered intervention start date, implemented for logistical reasons, did not appear to negatively impact data collection but allowed for more tailored engagement, as acknowledged by F6: “I also liked that you were really flexible… and that you kept giving us suggestions in the middle.”

The structured feedback sessions at weeks 4, 6, and 10 were well received. Participants appreciated the personalised reports and the opportunity to track progress. As F6 noted, “The suggestions during interim meetings were helpful, like pointing out a starch-heavy diet or needing more workouts,” and F1 shared, “After each meeting, we received feedback… So, improving that side was helpful.” These reflections highlight that the participants found the sessions constructive, practical, and supportive of behaviour change. Participants appreciated the personalised reports and the opportunity to track progress. One participant commented, “We do the grocery shopping together so we could sort of, I guess I’m getting a few more veggies fruits, and F2 has been having milk.” (M2), similarly, F2 commented, “Because you’re committed to doing it together… it definitely helps.” At the same time, some participants expressed a desire for more frequent feedback as suggested by F1. “I think if we had more sessions in between, we wouldn’t go off track.”

### 4.3. Adherence to the Intervention

A 100% retention rate was achieved by the eight couples over the 10-week intervention. All couples attended the four scheduled meetings, sent their data on time, and followed the timelines, further demonstrating high levels of adherence.

The couples-based approach was highlighted as a key factor in maintaining adherence, as F2 commented, “Doing this together kept us accountable and motivated.” Similarly, M7 noted, “I would have done it, but… it would have felt more like a chore. Making meals together but enforcing my needs on F7 would have been difficult.”

Most participants successfully tracked their diet and attempted to meet food group recommendations, which suggests these were realistic. As F7 noted, “We suggest meals to each other and keep each other accountable. ‘Does that have enough vegetables? Are we adding yoghurt?’ We try to keep the discussion lively.”

However, physical activity adherence was less consistent, particularly in increasing the intensity of physical activity levels. F4 highlighted that “In summer, we used to go around the lake here, but winter… by the time we come back home, it’s already dark, and I don’t feel like doing anything.” Similarly, M3 reflected, “It’s one of those things that easily gets pushed into the background when things get busy, and you have competing priorities.”

The nature of modern-day life sometimes limited participants’ ability to fully implement changes. As M8 noted, “Even if I was reminded through the study, it’s difficult to kind of get back on a track of eating healthy if they have other competing priorities that just make it really hard for me to cook or buy groceries and the like.”

### 4.4. Intervention Outcomes

#### 4.4.1. Changes in Anthropometric Measurements

The intervention aimed to promote changes in participants’ anthropometric measurements, with particular attention to BMI and waist-to-hip ratio, which are key indicators of metabolic health. Participants already within the healthy BMI range, such as M5 and M3, maintained stable values, reflecting the importance of recognising progress relative to individual baselines. Participants had mixed feelings about weight; for instance, M4 quoted, “It’s not just about losing weight, but about being healthy. The biggest takeaway for me was seeing the calories in one (fast food chain) burger. It made me realize how much I was consuming.”

Waist-to-hip ratio data similarly highlighted positive trends, even for participants who did not achieve the healthy thresholds (<0.85 for females and <0.9 for males) (see Table 3). The Wilcoxon signed-rank test indicated a statistically significant reduction in BMI (W = 21, *p* = 0.027) but no statistically significant change in waist-to-hip ratio (W = 55.5, *p* = 0.562) (Table 4). At baseline, three of the eight female participants and four of the eight male participants exceeded these thresholds. By the end of the intervention, reductions were observed in nine participants cases, such as F1, who reduced their ratio from 0.96 to 0.92, and M1, who reduced theirs from 0.95 to 0.88, entering the healthy range. These improvements are particularly encouraging given the short intervention duration.

Not all participants reached the healthy BMI range of 18.5–24.9 kg/m^2^ within the 10-week period, although several showed notable improvements (see Table 3). For example, M1 lowered their BMI from 25.4 to 24.7 kg/m^2^, and F4 reduced it from 28.6 to 27.9 kg/m^2^ (Table 3).

#### 4.4.2. Changes in Dietary Intake

The intervention included a focus on healthy eating, with participants encouraged to adopt healthier eating habits. Reflecting on the initial impact, M8 noted, “You don’t often think about eating more veggies or incorporating fruits. It was interesting to learn how to balance it out.” Motivation was sometimes linked to future family health, as F8 explained, “…if he (M8) wants his kids to have veggies, he needs to practice that.” Participants demonstrated notable dietary improvements, with increased vegetable and whole grain intake and a reduction in discretionary food consumption (Table 3). For vegetables, eight participants improved their intake during the intervention, though none met the recommended five serves by the end (see Table 3). Some participants noted that they did not realise the importance of dietary diversity. Many incorporated a variety of vegetables into their meals, reflecting a positive dietary shift. For instance, M6 shared, “I’ve reduced starchy vegetables and included more greens and reds in my diet,” and F5 noted, “The study made us more conscious of eating greens and veggies, so we added them to our shopping list. Our last seven-day diary should reflect more veggies than before.” However, some still struggled to incorporate vegetables consistently, as F8 also shared, “I gave him (M8) food garnished with raw baby spinach, and the lunchbox came back with only the green veggies left.”

In the fruit category, six participants increased their intake, but none met the recommendation of two servings. Grain intake showed the highest proportion of improvement, with 13 participants increasing their servings and 4 meeting the recommended six servings. The Wilcoxon signed-rank test confirmed a statistically significant increase in grain intake (W = 6.5, *p* = 0.002), indicating a meaningful shift in this food group. For other food groups, the test did not show statistically significant changes: vegetables (W = 46.5, *p* = 0.441), fruit (W = 14.0, *p* = 0.162), protein (W = 21.0, *p* = 0.502), and dairy (W = 44.0, *p* = 0.914) (see Table 4).

For protein-rich foods, 10 improved their protein intake, and 5 of those met the recommendation of 2.5 serves. Cultural practices often influenced adherence; for instance, M3 reflected, “The difficulty…is share plates, which are common in my Asian family.”

Dairy intake showed modest improvements, with six participants increasing their consumption, but none met the recommended three servings (Table 3). Limited preferences for dairy products were common barriers, so participants tried to think about milk substitutions as shared by M7 “We don’t really have any milk. So that I guess it’s thinking about, like other foods that we can get that calcium from.” Some used their future offspring’s health as a motivator, as noted by F2, “I know that women need calcium since before…But now, I am worried, it’s for the baby! So, I must have milk.”

Reducing discretionary food intake remained a common goal (Table 3) with 10 participants reducing their consumption, reflecting a trend toward healthier dietary habits. M3 shared, “Reminding me that my diet can always be cleaner and pointing out things I’m missing was helpful.” Similarly, F1 noted, “We used to drink a lot of Coca Cola, but now we have limited it.”

Traditional meal structures and food tracking systems did not always align with dietary recommendations or food composition databases adopted by current dietary assessment tools, making it difficult for participants to accurately log their intake. As F8 shared, “It was so hard for me to explain what a Haleem is to the app…… It’s just hard to log these things in apps designed for Western food.” F4 echoed this, noting, “Our food is different… Even though I thought it’s (the app) not very accurate for our diet. And also, we never have a concept of measuring food.” Despite these barriers, participants found ways to adapt. F2 explained,”Sometimes you just find something general that’s similar… but not that similar in a way.” M2 gave an example, saying, “Like a Thai red curry… you might find one in the app that’s less healthy than the fresh version we make, so we just put the ingredients in there ourselves.”

The couple-based intervention structure fostered shared responsibility and accountability in dietary changes. F7 described how meal planning became a collaborative activity: “We suggest meals to each other and keep each other accountable. ‘Does that have enough vegetables? Are we adding yogurt?’ We try to keep the discussion lively.” M2 highlighted how grocery shopping together reinforced healthier habits: “We do grocery shopping together, so we’ve been getting more veggies and fruits.” Overall, these findings highlight the feasibility of achieving dietary improvements within a 10-week intervention while acknowledging barriers to meeting recommended dietary guidelines. Participants’ reflections underline the role of couple-based support and cultural considerations in shaping dietary habits.

#### 4.4.3. Changes in Physical Activity

Sedentary behaviour was the primary focus of the physical activity data collected. While no significant increases in moderate or vigorous activity were reported, most participants demonstrated a reduction in sedentary habits by the end of the program (see Table 5). Quantitative data revealed that 75% of participants reduced their sedentary time. M2 commented, “I’m making a conscious effort to not be sedentary. I have a stand-up desk at work and need to use it more.” Participants noted that the awareness of the number of hours spent sitting helped. M5 noted, “I never thought I was spending at least 10 h a day just sitting and browsing. It gave me good insights into my lifestyle (and) my sedentary lifestyle leading to non-communicable diseases.”

Physical activity categories are based on the International Physical Activity Questionnaire (IPAQ): “Low” refers to activity levels below 600 MET-minutes per week, “Moderate” indicates activity levels of 600 MET-minutes per week or more, and “High” reflects either vigorous activity totalling at least 1500 MET-minutes per week or a combination of activities reaching 3000 MET-minutes per week. No participants in this sample were classified as “High.” MET stands for Metabolic Equivalent of Task.

Cultural differences were apparent in physical activity advise, as evidenced by F8’s comment: “I was shocked by the advice for pregnant and non-pregnant women. Back home, they tell you to sit and do nothing for nine months.” Maintaining a healthy lifestyle during difficult times was a challenge, as noted by F7: “When I’m stressed or feeling out of control, food and exercise drop off for me. I need to be mindful of that.”

An example of the couple’s dietary report and feedback and the participant’s individual data on BMI, waist-to-hip ratio, and changes in daily food group servings is available in Supplementary Tables S1–S3.

## 5. Discussion

This study demonstrates the feasibility of a couples-based 10-week lifestyle intervention designed to improve diet and physical activity whilst reducing the risk of Mets. By engaging both partners equally, the intervention capitalised on collaborative efforts and mutual accountability, demonstrating its potential to foster adherence to healthier behaviours [14]. The couples-based focus of our study underscores the importance of mutual involvement in achieving shared health goals, with previous studies noting that interventions lacking equal partner engagement often report lower retention and reduced adherence over time [16,49].

Adherence to our intervention was a key marker of its feasibility. The 100% retention rate, along with participants’ consistent engagement with program activities such as self-monitoring, scheduled meetings, and data submissions, underscores the structured and supportive nature of the intervention. Collecting body weight and waist-to-hip ratio at the start and end of the program provided clear indicators of engagement and adherence, consistent with literature highlighting the value of self-monitoring in lifestyle interventions [50]. These measures required active participant involvement and were crucial for monitoring progress over time [51]. Similarly, the completion of food diaries across multiple time points reflected participants’ sustained effort and investment in the intervention process [50]. Regular feedback sessions played a vital role in maintaining adherence, as participants valued the opportunity to track their progress and receive personalised advice. The couples-based structure was particularly impactful, fostering reciprocal motivation and shared accountability. This is consistent with previous research suggesting that dyadic interventions may enhance adherence through shared responsibility, whereas individually targeted programs often report lower engagement, especially over time [14,16,52]. Similarly, other couples based interventions have shown that partner involvement provides emotional and practical support that helps sustain adherence, especially during periods of low individual motivation [53].

Although only a 10-week intervention, the modest improvements across anthropometric outcomes, dietary intake, and physical activity that were observed highlight the feasibility of self-monitoring and the potential for short-term interventions to promote positive health changes, even when healthy ranges are not fully achieved. Notably, the Wilcoxon signed-rank test confirmed a statistically significant reduction in BMI (*p* = 0.027), suggesting that even within a short timeframe, meaningful changes in body composition can be achieved. However, no significant change was observed in waist-to-hip ratio (*p* = 0.562), indicating that improvements in fat distribution may require a longer intervention period or more targeted strategies.

Dietary improvements were a central outcome of the intervention, with participants reporting increased consumption of vegetables and grains alongside reductions in discretionary food intake. These changes reflect notable progress toward healthier eating habits, although most participants did not meet all dietary guidelines within the short period of the intervention. Similar findings have been observed in other short-term studies, where incremental progress often precedes full adherence to dietary recommendations [54,55]. Of concern, in 2022, was less than one in ten Australian adults (6.5%) met the recommendation for vegetable intake. Interestingly, the proportion of females meeting the dietary guideline for daily vegetable intake (9.8%) was approximately three times the proportion for males (3.0%). Collaborative activities, such as shared meal planning and grocery shopping, emerged as facilitators of dietary changes in our study, highlighting the value of partner involvement in fostering healthier habits [56,57]. These findings are consistent with previous qualitative studies of this kind [58]. Research has also shown that life stage transitions, such as preparing for parenthood, can prompt couples to renegotiate household food responsibilities and align health priorities [59].

Physical activity outcomes in our study were variable; while we observed success in reducing sedentary hours, similar improvements were not seen in meeting physical activity requirements. These findings align with prior research indicating that reducing sedentary behaviour is often more achievable than increasing higher-intensity activity levels, particularly within short-term interventions [49,54,60]. Participants in our study with lower baseline activity levels demonstrated the most improvement, reflecting the potential for significant gains among less active individuals. However, barriers such as work commitments, time constraints, and competing responsibilities limited participants’ ability to increase their physical activity levels [61], which is consistent with similar work of this kind [62]. Our qualitative data also revealed differences in motivation between partners and competing priorities that shaped physical activity participation, echoing findings from couples-based research that identified varying patterns of collaboration, independence, or conflict in physical activity dynamics [63]. These dynamics were also observed in our earlier qualitative work, where participants described how shared activity goals or mutual encouragement supported engagement, while mismatched motivation sometimes posed a barrier [21]. Future iterations of the intervention could provide more personalised strategies, such as maximising the use of incidental activities, flexible exercise plans or shared activity goals, to better address these challenges.

While our study demonstrated strong feasibility, several limitations must be acknowledged. First, the small sample size (eight couples) limits statistical power and generalisability. As a feasibility study, the aim was not to establish effectiveness, but the sample remains too small to draw conclusions for broader populations. Second, the absence of a control group restricts the ability to infer causality. Without a comparator, it is difficult to isolate the intervention’s effects from external influences. Third, the 10-week duration may be insufficient to assess sustained behaviour change or long-term outcomes. Longer follow-up periods are recommended in future studies. Another important limitation is the reliance on self-reported data for dietary intake, physical activity, and anthropometric measures. Despite training and standardised instructions, self-reporting is inherently subject to recall bias and misreporting. However, the results of the Wilcoxon signed-rank test address some of the limitations associated with small sample sizes and non-normally distributed data, providing a robust method for detecting meaningful changes in paired outcomes, as demonstrated in this study for BMI and grain intake. This test helped differentiate which health indicators were responsive to the intervention and which may require longer or more targeted strategies, offering a more nuanced understanding of its impact.

Additionally, the low-contact design, while intended to enhance scalability, may have limited the level of personalised support or engagement for some participants. Future research should include larger and more diverse samples, incorporate control groups, and consider longer durations. Where possible, the use of objective measures such as wearable devices or photo-based dietary logging tools could improve data accuracy [64,65]. Evaluating couples-based compared to individual approaches may also help identify the most effective models for different populations.

Finally, while the intervention was not specifically designed to explore cultural dietary practices, the cultural diversity of participants revealed cultural adaptation challenges in using standard dietary tracking tools. Most participants used the Easy Diet Diary app (version 6.0.28), which is based on the AUSNUT 2011–2012 database that primarily reflects Western dietary patterns [34,35,66]. Several participants reported difficulties logging traditional meals that lacked direct equivalents in the database. Although some adapted by entering individual ingredients, this approach was often imprecise and time-consuming. These issues align with broader concerns about the limited cultural responsiveness of current dietary assessment methods [67,68]. For the purpose of this study, which primarily focused on assessing adherence and feasibility, such limitations in dietary tracking were not critical to the main outcomes. However, they may have introduced inconsistencies in dietary intake data and should be addressed in future iterations. Expanding food composition databases and portioning systems to better reflect cultural diversity may improve the accuracy, relevance, and usability of self-monitoring tools, an important consideration in multicultural settings such as Australia.

Challenges were encountered, particularly in recruiting preconception couples, as the term ‘preconception’ was often misunderstood as implying fertility issues, which may have deterred some potential participants. Ensuring equal engagement of both partners also proved difficult, as differences in motivation levels and competing responsibilities sometimes limited participation. Similar challenges in engaging both partners have been reported by other researchers, primarily due to work and family commitments limiting participation in couples-based interventions [69]. This aligns with other qualitative research which found that many couples associate the term ‘preconception’ primarily with pregnancy planning or fertility, limiting its perceived relevance for those not actively trying to conceive [70]. Nevertheless, preconception health is increasingly recognised as a population-level concern rather than being limited to those actively trying to conceive, as optimising health before conception benefits not only future offspring but also parental health [71]. Reframing the intervention’s objectives to emphasise lifelong health benefits rather than reproductive intent could improve recruitment and engagement. Additionally, concern for future children can serve as a powerful motivator for lifestyle change, reinforcing the importance of engaging both partners in preconception health strategies [14]. The study focused on heterosexual couples; a design choice aimed to reduce biological variability in reproductive-related health factors while allowing for a targeted exploration of couple-based adherence dynamics. Future research could explore how these findings translate to a broader range of couple dynamics and reproductive contexts.

Beyond its immediate benefits to couples, this intervention holds promise for addressing the intergenerational transmission of Mets risk. Lifestyle interventions targeting both parents before conception can positively influence offspring health, reducing the risk of metabolic disorders in subsequent generations [66,72]. Further research on the epigenetic pathways that mediate these effects could assist in refining preconception interventions [66].

In summary, this study demonstrates the feasibility and potential for enhanced adherence of couples-based lifestyle interventions for reducing Mets risk. By engaging both partners equally, such interventions foster collaborative motivation, joint accountability, and sustainable behaviour change. These findings highlight the value of shared lifestyle factors and relationship dynamics in achieving notable health improvements, providing a promising pathway for multigenerational health promotion through preconception care.

### Implications for Clinical Practice

The findings of this study suggest that involving both partners in a couple-based lifestyle intervention can foster better adherence to health behaviour changes, particularly in preconception care. Clinicians could consider integrating couples-based strategies into lifestyle interventions, especially when aiming to reduce risk factors for Mets and other non-communicable diseases. By targeting both partners, healthcare providers can leverage the mutual support within couples to promote sustainable dietary and physical activity changes. This approach has the potential to improve both individual and shared health outcomes and reduce the intergenerational transmission of Mets risk.

## 6. Conclusions

To the best of our knowledge, this is the first study to test the feasibility of implementing a true couples-based lifestyle intervention aimed at improving diet and physical activity preconception to reduce the risk of Mets. The high retention rate and positive feedback from participants highlight the potential benefits of involving both partners in such interventions, particularly in terms of mutual support, accountability, and shared motivation to promote lasting behaviour change and intergenerational health. Participants reported improvements in dietary habits, such as increased vegetable intake, and reductions in sedentary behaviour, although challenges remained in increasing moderate-to-vigorous physical activity.

Quantitative analysis using the Wilcoxon signed-rank test provided further support for the intervention’s impact, revealing statistically significant improvements in BMI and grain intake. These findings suggest that even within a short 10-week timeframe, meaningful changes in key health behaviours and outcomes can be achieved, particularly when both partners are engaged. Future research should examine these promising findings utilising a larger sample size and a control arm, replacing self-reported with objective measures that acknowledge dietary cultural diversity over a longer time frame.

## Figures and Tables

**Table 1:** (**a**) Feasibility and adherence measures used in the couples-based lifestyle intervention. Includes both quantitative and qualitative metrics to evaluate participation, completion of tasks, and participant feedback. (**b**) Anthropometric, dietary, and physical activity outcome measures used to assess progress. Includes measurement tools and the thresholds used to define notable change.

(a)		
Outcome Measures	Description/Measurement Methods	
Feasibility	Feasibility was assessed by evaluating recruitment, retention, and the practicality of delivering the intervention to both members of a couple. Key outcomes included participation rates, completion of diaries and measurements, and participant feedback on intervention acceptability.	
Adherence	Adherence was assessed using:	
	• Quantitative Metrics: Completion of food diaries, submission of anthropometric data, and meeting attendance • Qualitative Insights: Interviews explored barriers, facilitators, and mutual support, such as how couples motivated each other to stay engaged in the intervention	
(**b**)		
**Outcome Measures**	**Description/Measurement Methods**	**Definition of Notable Change**
Body Mass Index (BMI)	Measured at baseline and endpoint to assess changes in body weight.	Progression toward the healthy BMI range (18.5–24.9 kg/m^2^).
Waist-to-Hip Ratio	Measured at baseline and endpoint to assess changes in central adiposity, indicating abdominal obesity.	Progression toward the recommended waist-to-hip ratio (<0.85 for females, <0.9 for males).
Dietary Intake	Assessed via food diaries focusing on the number of servings from key food groups (e.g., fruits, vegetables, grains, proteins). Participants were guided at baseline on how to record serves accurately.	Progression toward dietary recommendations (increase or reduction in servings as needed).
Physical Activity Levels	Measured using IPAQ and Borg’s scale, with a specific focus on changes in sedentary behaviour. Moderate and vigorous activities were combined.	Reduction in sedentary hours or progression toward higher physical activity levels.

Note: Participants already within the recommended range for each outcome measure were classified as “no change needed”.

**Table 2:** Sociodemographic characteristics and reasons for participation reported by couples enrolled in the intervention. Data includes age ranges, relationship status, and motivations provided at sign-up.

Participant	Gender	Age	Relationship Status	Work/Student Status	Reason for Participation
F1	Female	30–34	Married	Casual job	“Fitness, feel healthy physically and mentally, get prepared for a healthy pregnancy”
M1	Male	40–44		Working full-time	“To achieve healthy pregnancy along with a healthy baby”
F2	Female	30–34	Engaged	Working full-time	“To have motivation to do some exercises”
M2	Male	30–34		Working full-time	“Learn more about my overall health and habits I can improve”
F3	Female	20–24	Committed	Working full-time	“Learn more about my diet”
M3	Male	25–29		Working full-time	“Helping out with research”
F4	Female	25–29	Committed	Working full-time	“Weight loss”
M4	Male	25–29		Working full-time	“Weight loss”
F5	Female	30–34	Married	Working full-time	“Learn about healthy living facts”
M5	Male	35–39		Student	“Learning relevant information through the participation”
F6	Female	25–29	Married	Working part-time	“Common ground with partner for healthy lifestyle”
M6	Male	25–29		Working full-time	“Partner wants healthy lifestyle for us, fitness”
F7	Female	25–29	Engaged	Working full-time	“To learn more about my diet and physical activity habits and how I can improve them”
M7	Male	25–29		Working full-time	“I would like to learn more about my dieting habits and what I can do to improve them”
F8	Female	30–34	Married	A homemaker or stay-at home	“A better advice for a healthier living style”
M8	Male	30–34		Working full-time	“Fitness”

**Table 3:** Summary of changes in anthropometric outcomes and dietary intake across five food groups, relative to recommended thresholds. Improvements were observed even when guideline targets were not fully met.

Participants	BMI	Waist to Hip Ratio	Vegetable Intake	Fruit Intake	Grain Intake	Protein Intake	Dairy Intake
F1	✓	✓	✓	✓	✓	~	x
M1	✓	✓	✓	~	✓	✓	x
F2	~	~	✓	x	✓	~	x
M2	~	~	✓	~	✓	✓	x
F3	~	x	x	✓	✓	✓	x
M3	~	~	✓	x	✓	✓	✓
F4	✓	✓	x	x	✓	✓	✓
M4	✓	x	x	x	✓	✓	x
F5	✓	~	✓	~	x	✓	x
M5	~	✓	x	x	x	✓	✓
F6	~	~	x	✓	✓	x	x
M6	✓	✓	x	x	x	✓	x
F7	~	~	x	x	✓	x	✓
M7	~	~	✓	✓	x	✓	x
F8	✓	~	✓	x	✓	~	✓
M8	✓	~	x	✓	✓	~	✓

✓ Indicates improvements made by the participants. x Indicates areas where participants regressed or experienced a decline. ~ Indicates areas where no change was needed. Recommended BMI range = 18.5–24.9 kg/m^2^, recommended waist-to-hip ratio (WHR) = f ≤ 0.85, m ≤ 0.9. The recommended servings per day of each food group are as follows: vegetables = 5, fruit = 2, grains = 6, protein = 2.5, and dairy = 3.

**Table 4:** Summary of changes in anthropometric and dietary intake measures based on Wilcoxon signed-rank test results.

Outcome	Median (Start)	Median (End)	Test Statistic (W)	*p*-Value
BMI (kg/m^2^)	25.4	24.7	21	0.027
Waist-to-hip ratio	0.86	0.85	55.5	0.562
Vegetables	2.25	2.5	46.5	0.441
Fruit	1.00	1.0	14.0	0.162
Grain	3.88	5.5	6.5	0.002
Protein	2.88	2.5	21.0	0.502
Dairy	1.00	1.0	44.0	0.914

**Table 5:** Self-reported sedentary behaviour changes and endpoint physical activity classification of participants using the IPAQ.

Participants	Sedentary Habits	Category
F1	Improved	Low
M1	Improved	Low
F2	Improved	Low
M2	Improved	Moderate
F3	Improved	Moderate
M3	Improved	Low
F4	Regressed	Low
M4	Regressed	Low
F5	Improved	Low
M5	Improved	Low
F6	Improved	Low
M6	Improved	Low
F7	Regressed	Low
M7	Regressed	Low
F8	Improved	Low
M8	Improved	Moderate
Total improved	12 out of 16	

## Data Availability

The original contributions presented in this study are included in the article/Supplementary Material. Further inquiries can be directed to the corresponding author.

## Supplementary Materials

The following supporting information can be downloaded at: https://www.mdpi.com/article/10.3390/nu17122037/s1, Table S1: Couple Dietary Report and Nutritional Feedback sample; Table S2: Changes in BMI and Waist-to-Hip Ratio for Each Participant; Table S3: Changes in Daily Food Group Servings by Participants from Start to End of the Program.
