# Supplementary material for: Targeting Metabolic Syndrome with a Pre-Conception True-Couples-Based Lifestyle Intervention: A Pre-Post Mixed-Methods Evaluation
Source: Nutrients. 2025 Jun 18;17(12):2037. doi: 10.3390/nu17122037 (PMC12195852; doi:10.3390/nu17122037)
Supplement: Supplementary file 1 [file nutrients-17-02037-s001.zip › nutrients-3671879-supplementary.pdf]

Supplementary Table S1. Couple Dietary Report and Nutritional Feedback sample

**Food Groups (Recommended)**	**Female (Average intake)**	**Male (Average Intake)**
Vegetables (5)	2	1-2
Fruit (2)	1	2
Grains (6)	3.5	3
Protein (meat, eggs, poultry, fish, nuts, seeds, legumes) (2.5)	3	3
Dairy (and alternatives) (3)	0.5-1	1
Advice	Meeting protein needs<br>Increase vegetables particularly orange, red and green - we acknowledge some curries/stir fries may have more vegetables than is estimated<br>Increase calcium rich foods such as low-fat dairy or dairy alternatives such as fortified soy<br>A wholegrain breakfast cereal would be good to include or additional servings of fruit for breakfast	Meeting protein needs<br>Increase vegetables particularly orange, red and green - we acknowledge some curries/stir fries may have more vegetables than is estimated<br>Increase calcium rich foods such as low-fat dairy or dairy alternatives such as fortified soy<br>A wholegrain breakfast cereal would be good to include or additional servings of fruit for breakfast

Macronutrient Distribution
Female CHO - 72% (45-65); Fat – 14.4% (20-35); Protein – 13.6% (10-35).
Male CHO – 60% (45-65); Fat – 24.3% (20-35); Protein – 16.7% (10-35).
Please note: this is just a guide. I have noticed a number of potential discrepancies with recording (for example portions sizes that appear either too high or too low from a 'real' life perspective). Please emphasise the importance of trying as hard as possible to record food intake accurately and include brand names where possible.

Supplementary Table S2. Changes in BMI and Waist-to-Hip Ratio for Each Participant

**Participant**	**BMI – Start**	**BMI – End**	**% Change BMI**	**WHR – Start**	**WHR – End**	**% Change WHR**
F1	26.8	26.1	-2.61%	0.96	0.92	-4.17%
M1	25.4	24.7	-2.76%	0.95	0.88	-7.37%
F2	21.0	19.4	-7.62%	0.71	0.69	-2.82%
M2	23.5	23.8	+1.28%	0.81	0.80	-1.23%
F3	21.9	20.7	-5.48%	0.86	0.91	+5.81%
M3	23.8	24.3	+2.10%	0.80	0.81	+1.25%
F4	28.6	27.9	-2.45%	0.92	0.91	-1.09%
M4	26.3	25.9	-1.52%	0.94	0.96	+2.13%
F5	25.5	25.1	-1.57%	0.80	0.81	+1.25%
M5	22.5	22.5	0.00%	0.96	0.95	-1.04%
F6	23.1	23.8	+3.03%	0.66	0.70	+6.06%
M6	27.1	26.2	-3.32%	0.91	0.82	-9.89%
F7	21.9	21.0	-4.11%	0.78	0.80	+2.56%
M7	23.6	24.0	+1.69%	0.86	0.84	-2.33%
F8	35.8	35.3	-1.40%	0.80	0.76	-5.00%
M8	25.5	25.3	-0.78%	0.89	0.91	+2.25%
**Overall**			-1.68%			-0.58%

Summary:

- The mean percentage change in BMI across participants is -1.68%, suggesting a small overall decrease.
- The mean percentage change in WHR is -0.58%, indicating a very slight overall reduction.

Supplementary Table S3. Changes in Daily Food Group Servings by Participants from Start to End of the Program

Time point	Start	End	Start	End	Start	End	Start	End	Start	End
Participant	Vegetables	Vegetables	Fruit	Fruit	Grain	Grain	Protein	Protein	Dairy	Dairy
F1	1.5	3	1	1.5	4	6	2.5	2.5	1	0.5
M1	1.5	2.5	2	3	2	6	3.5	5	1	0.5
F2	1.5	2.5	1	1	2.5	3	2.5	2.5	1	1
M2	1.5	3.5	2	1.5	2	4	3.5	2.5	1	1
F3	1.75	1.5	0.5	1	3.75	5.5	4	3	1	0.75
M3	1	2.5	0.5	0.5	3.75	5.5	5.5	2.5	0.75	1.5
F4	3.5	3	1	0.5	1.5	6	2	3	1	1.5
M4	3.25	3	1.25	1	2.5	6	2	3	1.25	1
F5	2	3.5	2	2	4.5	4.5	3.5	3	1.25	0.75
M5	3.5	3.5	2.5	3	5	4.5	3.5	2.5	1	1.5
F6	3	2.5	0.25	0.5	3.5	5.5	2	2	2	0.5
M6	3.5	1.5	0.75	0.75	5.5	4.5	3	2.5	1.5	0.75
F7	3.5	2.5	0.75	0.75	4	5.5	3	3	1	1.5
M7	2.5	3.5	0.75	1	5	6.5	2.75	2.5	1.5	1.5
F8	1.75	2	1	1	4	5	2.5	2.5	0.5	1
M8	3.5	2	0	1	4.5	5	2.5	2.5	1.5	2

Recommended serves per day of each food group is as follows: Vegetables = 5, Fruit = 2, Grains = 6, Protein = 2.5, and Dairy = 3
